# The impact of implementing an allergic rhinitis clinical management pathway (AR-CMaP) in the community pharmacy

**DOI:** 10.1016/j.rcsop.2023.100340

**Published:** 2023-10-05

**Authors:** Rachel House, Vicky Kritikos, Biljana Cvetkovski, Janet Rimmer, Kwok Yan, Lynn Cheong, Jean Bousquet, Olga Lourenco, Sinthia Bosnic-Anticevich

**Affiliations:** a Quality Use of Respiratory Medicines Group, Woolcock Institute of Medical Research, Glebe, NSW, Australia; b Macquarie University, Macquarie Park, NSW, Australia; c Thoracic Medicine, St Vincent's Private Hospital, Darlinghurst, Australia; d Royal Prince Alfred Hospital, Camperdown, NSW, Australia; e Discipline of Pharmacy, University of Canberra, Canberra, Australia; f MACVIA-France, Contre les Maladies Chroniques pour un Vieillissement Actif en France, European Innovation Partnership on Active and Healthy Ageing Reference Site, Montpellier, France; g Faculty of Health Sciences and CICS—UBI, Health Sciences Research Centre, University of Beira Interior, 6200-506 Covilhã, Portugal

**Keywords:** Allergic rhinitis, Community pharmacy, Guideline, Self-select, Suboptimal management, Pharmacist

## Abstract

**Background:**

The Allergic Rhinitis Clinical Management Pathway (AR-CMaP) was developed to overcome the challenge of implementing current AR guidelines in the Australian community pharmacy practice and support pharmacists in optimally managing patients' AR.

**Objective(s):**

To evaluate the impact of AR-CMaP on patients' behaviour and pharmacists' needs in managing AR in the pharmacy.

**Methods:**

This study used a cross-sectional, pre-post study design in which the primary outcome was the appropriateness of medications purchased from community pharmacies in Australia. Patient data were collected before and after the implementation of AR-CMaP. Pharmacist needs were recorded before and after AR-CMaP training. Data were analysed descriptively.

**Results:**

Six pharmacies, 19 pharmacists and a total of 416 patients were included in the study; 206 pre-AR-CMaP implementation and 210 post-AR-CMaP implementation. Pre-AR-CMaP, 22.4% of patients purchased appropriate AR medication compared with 29.0% post-AR-CMaP implementation. Over half the patient cohort (52%) consulted a pharmacist pre-AR-CMaP and 37% consulted a pharmacist post-AR-CMaP implementation. Post-AR-CMaP, pharmacists reported increased awareness of barriers such as patients' lack of time, patients' perceptions about the pharmacist's role and patient choice to self-manage. Pharmacists also rated an increased desire to interact with other health care providers (HCPs) in caring for patients with AR.

**Conclusions:**

While there was a non-statistically significant increase in the proportion of patients purchasing optimal AR medication, AR-CMaP did empower patients to self-select their own medication without further detriment. Moreover, following the implementation of AR-CMaP, pharmacists developed a greater awareness of their role in AR management, exemplified by their increased desire to be actively involved in AR management and increased interaction with other HCPs. Future research needs to explore more effective tools to support pharmacists' clinical decision-making and target patients' self-selection of AR medications. This study highlights that there is an ingrained self-reliance of AR decision-making that has become a habit for people living with AR.

## 1 Introduction

Allergic rhinitis (AR) is a prevalent, chronic respiratory condition that is frequently mismanaged, causing extensive social and economic burden worldwide.^1^, ^2^, ^3^, ^4^ Misdiagnosis, continued exposure to environmental factors, and patient-related factors are key factors contributing to the high burden of AR; however, the ongoing concern worldwide is the suboptimal medication management of AR.^4^, ^5^, ^6^ Suboptimal use of AR medication is of particular concern in health care settings in which patients can self-select their own medication. In Australia, 70% of people purchasing AR medication in a community pharmacy, self-selected their AR medication^7^ and made suboptimal selections, 85% of the time.^5^ In this same cohort of people with AR, it was found that 92% of them had moderate-severe AR symptoms, of which 13% also had comorbid asthma, demonstrating the vulnerability of the population to increased risk of exacerbations and hospital admissions.^8^^,^^9^

While the responsibility of managing a chronic condition such as AR is shared between people with AR and health care providers (HCPs),^10^^,^^11^ what makes the management of AR more complex in everyday practice is the high value that people with AR put exclusively on their own decision-making.^12^ Previous research has explored the perspectives of people with AR, revealing that AR self-management is complex and evolves over time, often because of past experiences with HCPs and trials of different medications.^12^^,^^13^ Pharmacists and general practitioners (GP) have been identified as HCPs who are influential in AR management decision-making by people with AR, sometimes simply because of their accessibility when seeking a new treatment or advice when dissatisfied with their previous experience. It is through long periods of suboptimal therapies and mismanagement that people with AR develop low expectations about the efficacy of AR medications and rely on their own experiences. Therefore, it is imperative that the engagement between HCPs and people with AR is optimised.^12^, ^13^, ^14^, ^15^, ^16^

Although there is an obvious trend for people with AR to suboptimally self-select their medicines, it is evident that those with the highest burden of disease, do utilise a community pharmacy, where the pharmacist has access to AR guidelines.^17^ While it is recognised that there are several challenges in optimising AR management, the suboptimal management of AR raises several questions, most importantly, how can we bridge the gap between AR guidelines for pharmacists and the optimisation of AR management for their patients? A fundamental challenge is the lack of support for pharmacists to implement AR guidelines, with many cohorts worldwide not even being aware of the Allergic Rhinitis and its Impact on Asthma (ARIA) guidelines.^17^ Furthermore, while international guidelines aim to be considerate of local conditions and regulations, pharmacists may lack the organisational support required to understand how to do so, potentially contributing to a failure to implement.^18^^,^^19^

The Allergic Rhinitis Clinical Management Pathway (AR-CMaP) was specifically developed to support and overcome the challenges in the implementation of current AR guidelines in the Australian community pharmacy setting.^20^ This study aimed to evaluate the impact of AR-CMaP on patient behaviour and pharmacists' needs in managing AR in the community pharmacy.

## 2 Methods

This study used a cross-sectional, pre-post study design in which the primary outcome was the appropriateness of medication purchased by people with AR, which was the measurable and appropriate indicator of AR-CMaP implementation success.^20^ The secondary outcome was the proportion of people with AR who consulted a pharmacist as part of their AR medication selection process. This study was approved by the Human Research Ethics Committee (HREC) of the University of Sydney (2018/658).

### 2.1 AR-CMaP overview

AR-CMaP is the first, pharmacist-specific, guideline-informed, patient-centred implementation initiative, which supports pharmacists to facilitate AR diagnosis (where there is none), assess AR status and improve AR management through optimal medication selection and use over time.^20^ The development of AR-CMaP was based on the Promoting Action on Research Implementation in Health Services (PARIHS) implementation framework,^21^ AR clinical management pathways^17^^,^^22^ and empirical data on patient AR behaviour.^5^^,^^12^^,^^13^^,^^16^^,^^23^ The development of AR-CMaP is published in detail in Tan et al., ^20^

In summary, AR-CMaP consists of i) Pharmacists Needs Assessment (Appendix A in Tan et al., ^20^), ii) AR-CMaP webinar (60 min) to upskill pharmacists in using the latest ARIA guideline and online assessment of their knowledge and skills (Appendix B in Tan et al., ^20^), iii) an AR-CMaP implementation face-to-face workshop to address pharmacists' specific needs (determined by the Pharmacist Needs Assessment) and iv) AR-CMaP pharmacist and pharmacy patient management resources to increase patients' awareness of AR burden and encourage them to consult a pharmacist, (i.e, VAS rules, educational posters and shelf-talkers) (Appendix C and D in Tan et al., ^20^).

### 2.2 Pharmacy recruitment

The sampling frame for this study was service-oriented pharmacies, discount pharmacies (i.e., a pharmacy that operates on a low cost business model and often do not prioritise delivering pharmacy services) and hybrid pharmacies (loosely described as those with a mixed model of practice) in the Australian Capital Territory (ACT), Australia.

Pharmacies were invited to participate in the study with a letter of invitation and upon the return of an expression of interest to participate, pharmacy managers were required to consent in writing. All pharmacists employed within the enrolled pharmacies were invited to participate in the study and were provided with written study information. Written consent was obtained prior to pharmacist participation.

### 2.3 Patient recruitment

Patients, 18 years of age and over, who obtained AR medications for use in treating AR through self-selection or requesting medications from a pharmacist were eligible for study enrolment and were approached by a research assistant following purchase of their AR medication.

At the time of approach by the research assistant, patients were provided with verbal and written information (if requested). Verbal informed consent was required before study participation.

### 2.4 Study design and data collection

**Fig. 1:**
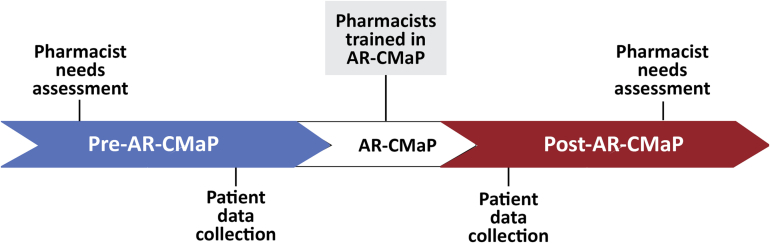
Study design and data collection

Fig. 1 summarises the study design for pharmacist and patient data collection and pharmacist AR-CMaP training which occurred during the Australian spring season in 2019 because that is when the highest proportion of people with AR visit the pharmacy. Pre-AR-CMaP data was collected to determine the AR medication selection processes within the pharmacy. This involved people with AR in the community pharmacy completing a baseline data collection form (Appendix E in Tan et al., ^20^), a research assistant-administered questionnaire which took 5 min to complete, following the purchase of an AR medication, in real-time, in the pharmacy. This was conducted over two week.

Following the pre-AR-CMaP data collection period, all participating pharmacists received training in AR-CMaP in their pharmacy. They were also provided with a suite of patient management resources for AR (i.e., Visual Analog Scale (VAS) rulers and AR education posters) to help engage and manage people with AR in the pharmacy.^20^

After AR-CMaP implementation, patient recruitment continued until the sample size was reached (as per the processes involved in collecting pre-AR-CMaP data) using follow up data collection form (Appendix F in Tan et al., ^20^). This data constituted post-AR-CMaP data.

Pharmacists were asked to complete a pharmacist needs assessment (Appendix A in Tan et al., ^20^) that took approximately 5–10 min, before receiving training in AR-CMaP and again at the end of the study after post-AR-CMaP data had been collected.

### 2.5 Data collection tools

Data for both patients and pharmacists were captured electronically using an online data collection and management website, Research Electronic Data Capture (REDCaP).

*Patient data collection form****:*** A research assistant-administered questionnaire (Appendix E and F in Tan et al., ^20^). was developed based on empirical evidence and the framework of ARIA guidelines.^18^ Data were collected across three domains:

i) Demographic characteristics (age, gender, method of product selection, diagnosis)
ii) Clinical symptoms for which the product was being purchased (nature, frequency, duration, and severity of symptoms [VAS score] and triggers), and
iii) Medication(s) purchased to treat AR symptoms.

*Pharmacist needs assessment****:*** The pharmacist needs assessment (Appendix A in Tan et al., ^20^). was developed using the ARIA 2018 framework for pharmacist-led management of AR.^7^^,^^18^^,^^24^ This questionnaire contained 41 items across three domains exploring: i) pharmacists' perceptions of their role in AR management, including AR diagnosis, assessment and medication management and self-management of people with AR, ii) pharmacists' perception of potential barriers to optimally address AR management in the pharmacy; and iii) pharmacists' perception of their interprofessional contact with other HCPs, including care for people with AR, patient-related perceived barriers and potential barriers relating to their perceived roles with doctors in AR management. For each item, pharmacists were required to respond on a scale of 1 (Strongly Disagree) to 5 (Strongly Agree) for items relating to Domains 1 and 3; and 1 = No Impact to 5 = High Impact for items relating to Domain 2.

### 2.6 Data management

*Patient data collection form****:*** Data was used to determine whether the choice of medication was optimal for people with AR. This was based on a previously reported method^5^ and involved a clinical panel of AR experts (consisting of one clinical researcher (RH), two clinical researchers and registered pharmacists with expertise in the management of AR (SBA and BC) and one practising respiratory physician and researcher (KY)) reviewing each questionnaire to confirm AR presentation and to determine the appropriateness of medication purchased.^5^ For people with AR, their AR were categorised as mild intermittent, mild persistent, moderate-severe intermittent and moderate-severe persistent, according to the ARIA guidelines^17^ (i.e., participants' reported frequency and extent of symptoms).

The appropriateness of AR medication purchased was based on the specific AR symptoms reported and categorised into:

a) Optimal (AR medication(s) purchased matched the VAS score and was consistent with the latest ARIA guideline recommendations^17^).
b) Suboptimal (AR medication(s) under-medicated for AR severity)
c) Inappropriate (medication(s) purchased not indicated for AR treatment)

*Pharmacist needs assessment*: The mean scores for each item (Likert Scale 1–5) were calculated.

### 2.7 Data analysis

Data collected from patient and pharmacist participants were exported from REDCaP into IBM SPSS version 28.0 (IBM Corporation, Armonk, NY), for analysis. Patient data collected through the baseline and follow up data collection forms were summarised using descriptive statistics. Data relating to patient demographics, AR (symptoms, seasonality, triggers), medication purchased and pharmacist interactions pre-and-post-AR-CMaP were compared using Chi-square tests for independence. Mean scores for each item in the pharmacist needs assessment were tested for normality, and the Wilcoxon signed-rank test was used for comparisons between paired data. A two-tailed significance level of 0.05 was used for all statistical procedures.

### 2.8 Sample size

A minimum of 400 patient participants (200 pre- and 200 post-AR-CMaP) were required to detect a doubling of the proportion of people with AR purchasing appropriate medication following the implementation of AR-CMaP (i.e. from 15% to 30%), at *p* = 0.8, *p* < 0.05, based on independent sample comparison and a cluster effect of 1.5.^5^ To take into account a positive AR diagnosis rate of 70% amongst people purchasing AR medications for AR, a total of 570 potential participants needed to be screened/approached.

## 3 Results

Seven pharmacies initially agreed to participate; five were service-oriented pharmacies, one hybrid and one discount pharmacy; the latter withdrew at an early stage citing issues with availability of staff. Therefore, six pharmacies and 19 pharmacists were enrolled, with data collection occurring between September 2019 and November 2019. Patient data were collected as research staff were available during normal pharmacy business hours.

**Fig. 2:**
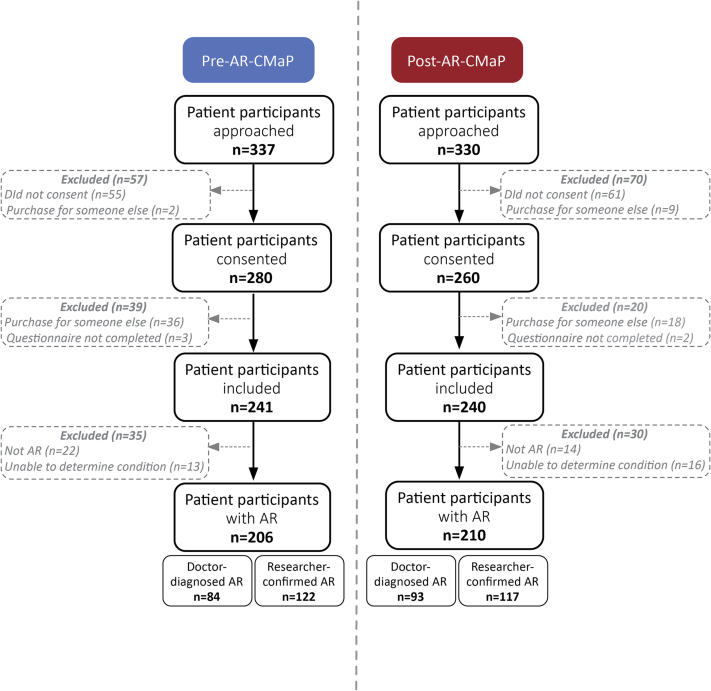
Flow chart of survey response rates for pre-and-post-AR-CMaP.

Fig. 2 summarises the patients recruited for this study. A total of 667 patients were approached and screened; 206 and 210 patients were identified with AR at pre-and-post-AR-CMaP respectively. There was no significant difference between the proportion of people with a doctor diagnosis of AR between the pre-AR-CMaP and and-post-AR-CMaP groups (41% (84/206) versus 44% (93/210)) (Chi-square test, *n* = 416, *p* = 0.489) (Fig. 2).

### 3.1 Demographics and Clinical Characteristics of patient participants with AR

There were no significant differences in the overall demographic and clinical characteristics of people with AR recruited into the study pre-AR-CMaP and post-AR-CMaP. The majority (61%) of participants were aged over 40 years and over 98% of this cohort were experiencing moderate-severe AR (Table 1). The most common symptom experienced by people with AR was sneezing.

**Table 1:** Demographics and Clinical Symptoms of participants pre-AR-CMaP and-post-AR-CMaP (*n* = 416).

	Pre-AR-CMaP n/N (%) (*N* = 206)	Post-AR-CMaP n/N (%) (*N* = 210)	*p*-value
**Age**			
>40 years old	125 (61%)	129 (61%)	0.920
**Gender**			
Female	128 (62%)	135 (64%)	0.685
**Symptoms**			
Sneezing	156 (76%)	151 (72%)	0.435
Blocked nose	106 (52%)	100 (48%)	0.492
Runny nose	137 (67%)	130 (62%)	0.358
Itchy nose	91 (44%)	82 (39%)	0.320
Itchy/Watery eyes	142 (69%)	151 (72%)	0.521
Wheeze ± asthma	48 (23%)	57 (27%)	0.430
**Symptoms flare up**			
Spring	153 (74%)	170 (81%)	0.126
Autumn	12 (6%)	7 (3%)	0.248
Summer	15 (7%)	22 (11%)	0.302
Winter	5 (2%)	8 (4%)	1.000
**Trigger**			
Identified symptoms' trigger	178 (86%)	172 (82%)	0.288
Pollen	168 (82%)	151 (72%)	0.021⁎
House Dust Mites	51 (25%)	44 (21%)	0.414
Animal Dander	40 (19%)	40 (19%)	1.000
Moulds	9 (4%)	11 (5%)	0.679
**Classification**			
Mild Intermittent	4 (1.9%)	2 (1.0%)	0.446
Mild Persistent	1 (0.5%)	0 (0.0%)	0.495
Moderate-Severe Intermittent	100 (49%)	106 (51%)	0.696
Moderate-Severe Persistent	101 (49%)	102 (49%)	1.000

⁎ Statistically significant (Chi-square test for independence, p < 0.05).

### 3.2 Medication selection

Table 2 summarises the medications purchased by people with AR. A total of 360 people with AR purchased monotherapy pre-AR-CMaP (180/205, 88%) and post-AR-CMaP (180/210, 86%). The most common monotherapy purchased both pre-AR-CMaP and post-AR-CMaP was oral antihistamines (OAH) followed by intranasal corticosteroids (INCS) (Table 2). The most common dual therapy purchased pre-AR-CMaP and post AR-CMaP was OAH + INCS. There was a statistically significant increase in the proportion of people with AR purchasing intraocular antihistamines (IOAH) post-AR-CMaP compared to pre-AR-CMaP (10% vs. 4%, *p* = 0.02) (Table 2).

**Table 2:** AR Medications purchased pre-AR-CMaP and-post-AR-CMaP (n = 416).

Medicine/Product purchased	Pre-AR-CMaP n (%) (*n* = 205)⁎⁎	Post-AR-CMaP n (%) (*n* = 210)	p-value
**Monotherapy**			
Oral Antihistamines (OAH)	113 (55.1)	105 (50.0)	0.434
Intranasal Antihistamines (INAH)	1 (0.5)	2 (1.0)	1.000
Intranasal Corticosteroids (INCS)	42 (20.4)	41 (19.5)	0.902
Intranasal Decongestants (IND)	7 (3.4)	4 (1.9)	0.377
Saline irrigation	8 (3.9)	4 (1.9)	0.256
Intraocular Antihistamines (IOAH)	9 (4.4)	22 (10.5)	0.020⁎
Ipratropium	0 (0.0)	1 (0.5)	0.495
Montelukast	0 (0.0)	1 (0.5)	0.495

**Combined Therapy**			
OAH + INAH	1 (0.5)	0 (0.0)	0.495
OAH + INCS	13 (6.3)	11 (5.2)	0.526
OAH + IND	1 (0.5)	1 (0.5)	1.000
OAH + Saline	0 (0.0)	3 (1.4)	0.499
OAH + IOAH	6 (2.9)	2 (1.0)	0.172
INAH + INCS	2 (1.0)	5 (2.4)	0.449
INAH + INCS + IOAH	1 (0.5)	0 (0.0)	0.495
INAH + INCS + OAH	0 (0.0)	1 (0.5)	0.495
INCS + IND	0 (0.0)	2 (1.0)	0.499
INCS + Saline irrigation	1 (0.5)	0 (0.0)	0.495
INCS + IOAH	0 (0.0)	3 (1.4)	0.499
IOAH + Saline irrigation	0 (0.0)	1 (0.5)	0.495
IND + Saline irrigation	0 (0.0)	1 (0.5)	0.495

⁎ Statistically significant difference (Chi-square test for independence, p < 0.05).

⁎⁎ one patient was using steroid tablets.

**Fig. 3:**
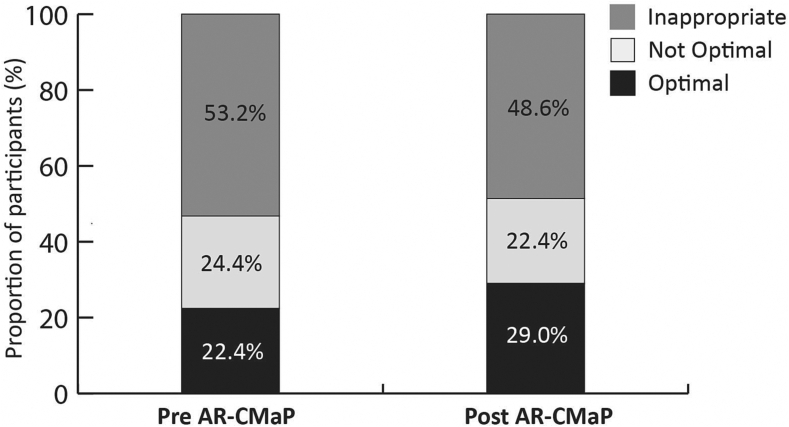
Appropriateness of medication purchased by people with AR pre AR-CMaP (n=205)* and post-AR-CMaP (n=210).*one patient was using steroid tablets therefore the appropriateness of treatments could not be determined.

Fig. 3 summarises the appropriateness of medication purchased by people with AR pre-AR-CMaP and-post-AR-CMaP. The increase in the proportion of optimal medications purchased from pre-AR-CMaP to post AR-CMaP was not statistically significant (22.4% versus 29.0% respectively, Chi-square test for independence, *p* = 0.185, *p* = 0.511 and *p* = 0.306 respectively).

There was no significant difference in the proportion of people with AR and wheeze/asthma, who purchased INCS pre-AR-CMaP compared with post-AR-CMaP (27.7% versus 28.1% respectively, Chi-square test, *n* = 415, *p* = 1.000).

There was a statistically significant increase in the proportion of people with AR with itchy/watery eye symptoms, who purchased optimal medications pre-AR-CMaP compared with post-AR-CMaP (11.3% vs 20.5%; Chi-square test, n = 415, *p* = 0.038).

There was no significant difference between the proportion of people with AR purchasing optimal medication pre-AR-CMaP versus post-AR-CMaP, when subset analysis based on pharmacy type was conducted comparing service-oriented and hybrid-oriented pharmacy models. (26% versus 24.5% respectively, Chi-square test, n = 415, *p* = 0.821).

### 3.3 Consultations with the pharmacist

There was a statistically significant decrease in the proportion of people with AR who consulted a pharmacist pre-AR-CMaP compared to post-AR-CMaP (52% versus 37% respectively; Chi-square test, *n* = 416, *p* = 0.004). Of those who consulted the pharmacist, there was no statistically significant difference between the proportion of people with AR who purchased optimal medication after consulting with a pharmacist pre-AR-CMaP compared to post-AR-CMaP (61% versus 53% respectively, Chi-square test, *n* = 184, *p* = 0.240).

### 3.4 Pharmacist's needs assessment

The pharmacist needs assessment was completed by 19 pharmacists pre-AR-CMaP and eight pharmacists post-AR-CMaP implementation (44% response rate of pre-AR-CMaP). Table 3 summarises the paired results of the eight pharmacists who completed the needs assessment both pre-and-post-AR-CMaP.

**Table 3:** **Pharmacist needs assessment responses pre-and-post-AR-CMaP implementation**.

	Pre-AR-CMaP (Mean ± s.d.) *n* = 8	Post-AR-CMaP (Mean ± s.d.) n = 8	p-value@
Domain 1	The role of the community pharmacists in the management of allergic rhinitis:		
	DIAGNOSIS*		
1. Differential diagnosis of allergic rhinitis	4.63 ± 0.517	4.50 ± 0.534	0.317
2. Evaluating symptoms experienced by patients with allergic rhinitis	4.88 ± 0.353	4.75 ± 0.462	0.564
3. Identifying the extent of the impact of symptoms on quality of life to determine the severity of symptoms	4.75 ± 0.462	4.63 ± 0.517	0.564
4. Investigating triggers/factors that worsen patients' symptoms	4.75 ± 0.462	4.50 ± 0.534	0.157
5. Referring to doctors for complicated rhinitis	5.00 ± 0.000	4.88 ± 0.353	0.317
6. Check for asthma in patients with allergic rhinitis	4.50 ± 0.534	4.38 ± 0.517	0.564
	MEDICATION MANAGEMENT*		
7. Recommending individualised medications for each patient with allergic rhinitis	4.63 ± 0.517	4.63 ± 0.517	1.000
8. Identifying inappropriate use of oral antihistamines	4.25 ± 0.707	4.63 ± 0.517	0.257
9.Educating on long term use of decongestants	4.88 ± 0.353	4.75 ± 0.462	0.564
10. Identifying poor adherence with maintenance treatment	4.50 ± 0.534	4.50 ± 0.534	1.000
11. Counselling on allergen-specific immunotherapy	3.50 ± 1.069	3.63 ± 0.916	0.564
12. Managing allergic rhinitis in children ages 5–16 years	4.25 ± 0.707	4.00 ± 0.534	0.157
13. Managing allergic rhinitis in elderly	4.25 ± 0.462	4.25 ± 0.462	1.000
14. Reviewing the effectiveness of medications over time	4.50 ± 0.534	4.38 ± 0.517	0.564
15. Identifying circumstances in which GP referral is required	5.00 ± 0.000	4.75 ± 0.462	0.157
	PATIENTS' SELF-MANAGEMENT*		
16. Ensuring that each patient has an allergic rhinitis treatment plan	4.00 ± 0.755	4.25 ± 0.462	0.157
17. Identifying triggering factors and establish minimisation strategies	4.50 ± 0.534	4.25 ± 0.707	0.317
18. Educating patients on how to recognise when allergic rhinitis worsens and how to take action when it does	4.63 ± 0.517	4.50 ± 0.534	0.317
19. Educating patients on how to self-monitor symptom control	4.63 ± 0.517	4.50 ± 0.534	0.317
20. Counselling about the impact of allergic rhinitis on quality of life	4.50 ± 0.534	4.50 ± 0.534	1.000
21. Demonstrating proper intranasal technique when first prescribed an intranasal device	4.88 ± 0.353	4.88 ± 0.353	1.000
22. Monitoring the patients' intranasal technique on a regular basis	3.88 ± 1.457	4.38 ± 0.517	0.257
23. Explaining the link between allergic rhinitis and asthma	4.25 ± 0.707	4.13 ± 0.353	0.564

**Domain 2**	**Pharmacist perceived barriers to optimally addressing allergic rhinitis management in the pharmacy:**		
	PHARMACIST RELATED**		
24. Lack of time by the pharmacist	3.00 ± 1.069	3.75 ± 0.462	0.098
25. Lack of resources (placebo devices, leaflets)	3.00 ± 0.534	2.75 ± 1.035	0.527
26. Lack of suitable private area in the pharmacy	2.13 ± 0.640	2.13 ± 1.125	0.891
27. Pharmacists' perception that it is not their role	1.63 ± 1.060	1.63 ± 1.060	0.891
28. Trying not to “overstep” the role of the doctor	1.88 ± 0.640	2.13 ± 1.246	0.414
29. The conflict between professional and commercial interests	1.50 ± 0.755	1.63 ± 1.060	1.000
30. No financial incentive	1.13 ± 0.353	1.88 ± 1.125	0.059
31. Language barriers	2.88 ± 0.834	3.13 ± 0.834	0.317
	PATIENT RELATED**		
32. Lack of time by the patients	3.13 ± 0.834	4.00 ± 0.755	0.035^^^
33. Patients' perception that it is not the pharmacists' role	2.50 ± 0.925	3.00 ± 0.755	0.102
34. Patients' health belief	3.00 ± 0.534	3.38 ± 0.744	0.083
35. Patients' lack of allergic rhinitis knowledge	3.75 ± 0.886	3.63 ± 0.744	0.564
36. Patients choose to self-manage	3.25 ± 1.035	4.00 ± 0.755	0.034^^^
37. Patients' perception act they are well cared for by the doctor	3.13 ± 0.991	3.00 ± −0.755	0.705

**Domain 3**	Interprofessional Relationship and interaction*		
38. I have good interprofessional contact with other health care professionals with regards to care of my patients with persistent allergic rhinitis	3.38 ± 1.060	3.75 ± −0.886	0.180
39. I would like to have more contact with other health care professionals with regards to the care of my patients with persistent allergic rhinitis	3.38 ± 0.744	4.13 ± −0.640	0.034^^^
40. I would like to have more interactions with health care professionals because of perceived patient-related barriers	3.75 ± 0.707	3.75 ± −0.707	1.000
41. I would like to have more interactions with health care professionals because of barriers relating to the perceived roles of doctor and pharmacist in allergic rhinitis management	3.50 ± 0.925	3.88 ± −0.640	0.257

* Likert Scale: 1 = Strongly Disagree |2 = Disagree |3 = Neither Disagree/Agree |4 = Agree |5 = Strongly Agree; ** Likert Scale: 1 = No Impact |2 = Slight Impact |3 = Moderate Impact |4 = Considerable Impact |5 = High Impact.

^ Statistically significant difference (Chi-square test for independence, p < 0.05).

@ Wilcoxon signed-rank test used to compare the paired sample analysis on n = 8 pre-and-post-AR-CMaP pharmacist data.

Responses across 23 items in Domain 1 indicated that overall, pharmacists agreed/strongly agreed that they had a role in AR diagnosis, medication management and supporting patient self-management. There was no statistically significant difference in pharmacists' responses pre-AR-CMaP versus post-AR-CMaP with regards to Domain 1 (Table 3).

Of the 8 potential pharmacist-related barriers in Domain 2, the 2 major barriers were lack of time by the pharmacist and language barrier. There was no statistically significant difference in pharmacists' responses pre-AR-CMaP versus post-AR-CMaP with regards to Domain 2 (Table 3).

Of the 6 potential patient-related barriers in Domain 2, the major barrier identified by pharmacists was patients' lack of AR knowledge. There was a significant increase in agreement of pharmacists that patients' lack of time and patients' choice to self-manage were barriers to addressing AR properly in the pharmacy, post-AR-CMaP compared with pre-AR-CMaP (Table 3).

Amongst the interprofessional relationship and interaction in Domain 3, there was a statistically significant increase in agreement of pharmacists wanting to work with other health care providers around AR management post-AR-CMaP compared with pre-AR-CMaP (Table 3).

## 4 Discussion

This study builds on the previous extensive work of the research team on the suboptimal management of AR in the community pharmacy. It once again confirms that a high proportion of people seeking AR medication do not have a doctor diagnosis, suboptimally self-select their medicines and even a highly specialised education and training program for community pharmacists, does little to address these major issues. This study aimed to improve AR management through the implementation and evaluation of AR-CMaP, a community pharmacy-specific AR intervention, responsive to the high self-selection health behaviour of people with AR who visit the community pharmacy and aimed at increasing the purchase of appropriate medication. As noted above, the results of this study indicate that AR-CMaP did not result in an overall increase in the proportion of people with AR purchasing optimal medication, however it did increase the proportion of people with AR and ocular symptoms purchasing optimal medication. There was also an increase in the proportion of people with AR who self-selected their own AR medication, without a decrease in those purchasing optimal medication, hence, AR-CMaP did appear to support the process of self-selection. In terms of assessing pharmacists' needs, AR-CMaP seemed to highlight the more complex nature of managing AR in the community pharmacy, with pharmacists being more aware of the need for more time and the importance in overcoming patient self-management as critical barriers, while expressing a desire to work more closely with other health care providers in managing AR.

People with AR in this study showed both similarities and differences to previous AR populations studied within community pharmacy. Similarly, there was a high proportion (98%) of people with moderate-severe AR in this study.^7^ However, the symptom profile of people with AR in this study was different than those in other relevant studies in that sneezing followed by itchy/watery eyes and runny nose were the most frequently reported symptom, rather than nasal symptoms alone (blocked nose, runny nose, sneezing) which has previously been identified as the most frequent reported symptom.^7^ There was also almost twice as high a proportion of people with AR and coexisting asthma/wheeze in this study compared with others.^7^ These comparisons imply that people with AR in this study are of a different phenotype.^26^ Research has identified that there are up to 6 AR phenotypes classified by different biomarkers and the presence, absence or co-existing presence of nasal, ocular and/or lower respiratory symptoms.^26^ Given the fact that this study, has for the first time, identified potentially different clusters in the community pharmacy setting, it is important to consider the different AR phenotypes and their implications for future research and practice.

A key feature of the AR-CMaP was addressing the high proportion of AR medication self-selection. For this reason, AR-CMaP contained both a consumer/patient component, while also specifically addressing the known perceptions and experiences of people with AR and their medication-selection behaviour.^23^^,^^5^, ^27^, ^28^, ^29^, ^30^, ^31^ Within AR-CMaP, a few patient-specific tools were developed for display in the pharmacy, for the purpose of: i) aiding better AR self-assessment and self-selection of medications and ii) driving a consultation with a pharmacist for those with more severe AR or who had experienced treatment failure in the past. The impact of these tools on overall purchase of optimal medication, while trending towards an increase (23% to 29%), was not statistically significant. Neither was there an increase in the purchase of appropriate medication for the cohort with coexisting asthma, despite the critical importance of these individuals needing optimal medication.^33^ What was obvious however was that for people with AR who were experiencing itchy/watery eyes, there was a significant improvement in the purchase of optimal medication, once again, drawing our attention to a particular AR phenotype (i.e., involves both nasal and ocular symptomatology).

This study showed a relatively high level of pharmacist engagement pre-AR-CMaP implementation (i.e., over half the cohort (52%) consulted the pharmacist for AR). This is noticeably higher than the proportion of people with AR consulting with the pharmacist in previous studies.^5^ However, post-AR-CMaP, patient self-selection of AR medication increased, potentially providing further insights into this cohort of pharmacies and patient behaviours around AR management. Firstly, all pharmacies were either service-based or hybrid-based pharmacies. This would suggest that maximum patient engagement around AR is already happening within these pharmacies. Secondly, this perhaps reflects the fact that there was an increase in patient independent medication decision-making as the additional tools that were provided as part of AR-CMaP, empowered the patient to make their own medication selection. This they did with no further detriment, though not ideal, as the purchase of optimal medications still remained low. Thirdly, given that follow up data were collected later in the pollen season, this may reflect the medication-taking behaviour of people with AR throughout the pollen season. From other research, it can be hypothesised that, although some people with AR value pharmacists' opinion and advice, they may only seek pharmacist recommendations early in the pollen season and did not see the need for a review later on.^12^^,^^13^ This may also reflect/highlight the challenges of long-term monitoring of AR in the community and give the community perception that a review is not warranted nor common practice in a community pharmacy setting.^13^^,^^29^

When it came to the pharmacists and perceptions of their role in AR management, overall and consistently, from the outset (i.e., pre-AR-CMaP), pharmacists rated their role highly across all items from diagnosis to referral and everything in between. This did not change post-AR-CMaP and suggests a desire to be actively involved in AR management. Hence, when it comes to barriers to implementing AR management in the pharmacy, pharmacists essentially were either neutral or rated pharmacy-related barriers lowly (i.e., not as important as patient factors). However, post-AR-CMaP, pharmacists reported higher agreement with three patient-related factors: patients' lack of time, patients' perceptions about the pharmacist's role and patient choice to self-manage. This suggests that either pharmacists became more aware or found it more difficult to engage with the patient post-AR-CMaP and this is reflected in the decreased proportion of people with AR who consulted with the pharmacist.

Pharmacists also registered an increased desire for interacting with other HCPs in caring for patients with persistent AR; this suggests better identification of referral pathways and communication with other HCPs should be considered in the future. While international frameworks for AR management recognise the care pathway, connecting patients in pharmacy to GP and then to specialists,^35^ there is no formal pathway for this to occur or criteria/screening tools for pharmacists to identify and refer a patient to a HCP. It is obvious that poorly controlled AR, despite using optimal AR medication should be a trigger for referral, however tools are needed to be considered for identification of high-risk patients. In this study, over 80% of people with AR identified spring as a trigger for their AR and over 70% specifically noted pollens; all of whom reported moderate-severe AR symptoms. According to the guidelines, they should be taking INCS at the very least, or some might be candidates for immunotherapy. However, without a clear referral pathway, this would be a challenge for pharmacists to identify without appropriate criteria/screening tools.^36^

### 4.1 Limitations

There was no control group in this study due to the lack of resources for recruiting people with AR during high AR flare-up season. Hence a pre-post study design was implemented, which did not include matched pairs. Further, we can now hypothesise that people with AR who visited the community pharmacy pre-AR-CMaP may have a different phenotype to those who visited the pharmacy post-AR-CMaP as they were less likely to report pollen as a trigger to their AR. It would have therefore been important to recruit/sample our population based on AR phenotypes. Another limitation of this study is the lack of feedback post-AR-CMaP from people with AR. Prior to the implementation of this study, the process of getting feedback from people with AR was considered, however, being an implementation study, the burden of research data collection and the potential for this to hinder participation of people with AR was weighed against the benefits associated with getting feedback from people with AR. It was determined that participation was the priority objective. Therefore, moving forward, it will be critical to get feedback from people with AR, especially given the increased self-selection of AR medication observed in this study post-AR-CMaP. A last limitation to this study was that we were not able to apply all current global guidelines^37^ to this study as Australian guidelines were used to match the local context.

## 5 Conclusions

AR-CMaP appeared to empower people with AR in self-selecting their own AR medication with the tools provided post-AR-CMaP, with a slight improvement in the appropriateness of optimal medication purchased and improvement only amongst individuals with the ocular symptom AR phenotype. This highlights the challenges of AR management in the community and points us to several important considerations for the future. Firstly, improved care pathways for AR in the community pharmacy are desperately needed. Secondly, care pathways need to address the desire of pharmacists to engage with patients and other HCPs but must better support the needs of people with AR who are clearly determined to manage AR more independently. Lastly and perhaps most urgent, this study, truly highlights the necessity for greater understanding of the role of the patient in AR management. In particular, patient health behaviours and all aspects of their perceptions and experiences which leads patients to AR self-management. Only once the perspective, experiences and challenges of people with AR have been truly understood, the needs can then be addressed within the context of community pharmacy. Until this is achievable, it will still be a struggle to reduce the burden of AR in the community.

## Funding

This study is partly supported through an Investigator-Initiated Woolcock-Mylan Partnership Grant 001984.

## Declaration of Competing Interest

RH, BC, LC, OL declare no conflict of interest. VK has received honoraria from AstraZeneca, GlaxoSmithKline and Pfizer. JR declares no conflict of interest, has received sponsorship from GlaxoSmithKline, AstraZeneca, Sanofi, Stallergenes. KY has received honoraria for speaking and consulting from AstraZeneca, Boehringer Ingelheim, GlaxoSmithKline, Meda, Mundipharma and Pfizer. JB reports personal fees from Chiesi, Cipla, Hikma, Menarini, Mundipharma, Mylan, Novartis, Purina, Sanofi- Aventis, Takeda, Teva, Uriach, other from KYomed-Innov, outside the submitted work. SBA is a member of the Teva Pharmaceuticals Devices International Key Experts Panel, has received research support from Research in Real Life, has received lecture fees and payment for developing educational presentations from Teva, GlaxoSmithKline, AstraZeneca and Mundipharma; and has received Honoria from AstraZeneca, 10.13039/100001003Boehringer Ingelheim, GlaxoSmithKline, for her contribution to advisory boards/key international expert forum.
